# The Sins of the Fathers

**Published:** 1920-02-14

**Authors:** 


### The Sins of the Fathers.

The People's League of Health, under Miss Olga Nethersole's energetic organisation, is carrying on its work with enthusiasm and wisdom. Miss Nethersole, addressing a Leicester meeting the other day, dealt with the startling statistics regarding preventable infectious diseases collected by the League, and she gave her description of the true meaning of tragedy as not the sufferings from our own actions, but the results of the actions of others which descend upon us. This has always seemed the strong point in the contentions of those who advocate prophylactic measures with regard to venereal disease, and, though the whole subject is one of deep complexity, there seems a broad reasonableness worthy of consideration in the resolution passed at a meeting (presided over by the Lord Mayor) of the Society for Prevention of Venereal Disease. According to this, it is obvious that the best way of avoiding venereal disease is to abstain from promiscuous sexual intercourse. It is, however, certain that a large number of persons continue, in spite of moral teaching, to expose themselves to risk, and so to incur and spread disease amongst the community, the chief sufferers being women and children. Venereal disease has become a menace to national health and prosperity, and in view of the proved fact that infection can be prevented by means of self-disinfection, if properly applied, immediately after exposure to risk, it is necessary to instruct the public as to :—(*a*) The vital importance of self-disinfection at the times of exposure to risk as a preventive of venereal disease; and (*b*) the methods of application.

To return to Miss Nethersole, she declares that there are a million children needing serious immediate help in England and Wales, and she wisely says that of still greater importance is the responsibility for the birth of normal healthy children in the future. She hopes to see in the near future regulations preventing women from working in a factory or shop for a month before childbirth and a month afterwards. In this, as in all other matters of health and progress, the public conscience must be awakened.

